# Digital CBT-I versus stepped-care CBT-I to prevent depression one year later

**DOI:** 10.1192/j.eurpsy.2023.303

**Published:** 2023-07-19

**Authors:** P. Cheng, D. Kalmbach, C. Sagong, C. Fellman-Couture, C. Drake

**Affiliations:** Sleep Research, Henry Ford Health, Novi, United States

## Abstract

**Introduction:**

Insomnia is a robust risk factor for depression. Treating insomnia with digital CBT-I (dCBT-I) has been shown to prevent future episodes of depression; however, remission rate of insomnia following dCBT-I is lower compared to face-to-face CBT-I (fCBT-I), which may reduce the effect on depression prevention. A stepped-care model can optimize care by starting with a least resource intensive intervention (step 1: dCBT-I) and stepping-up non-remitters to specialized treatment (step 2: face-to-face CBT-I).

**Objectives:**

This study examined the efficacy of a stepped-care approach to prevent depression.

**Methods:**

1018 individuals with DSM-5 insomnia and no depression were randomized into two conditions at step 1: dCBT-I (n=613), or an online sleep education control (n=624). Participants in the dCBT-I condition who did not show remission for insomnia (ISI>9) were further randomized to either face-to-face CBT-I (n=103) or sleep education (n=104). Rates of clinically significant depression (moderate severity and above) was assessed at one-year follow-up.

**Results:**

Insomnia remission rates were higher in the dCBT-I group (40%) compared to the control group (22%). Those who did not remit following step-1 dCBT-I showed step-2 insomnia remission rates of 75% following fCBT-I compared to 38% following the step 2 control.

At one year follow-up, the incident rate of clinically significant depression was 2.4 times higher in those who received control (13.2%) compared to fCBT-I (5.5%) at step 2. Depression rate was 10.1% in those who did not receive dCBT-I at step-1.

**Conclusions:**

Preliminary evidence from this study provide supported that a stepped-care approach may produce greater protection against incident depression than dCBT-I alone.

**Disclosure of Interest:**

None Declared

